# When Multiple Objective Measures of Medication Adherence Indicate Incongruent Adherence Results: An Example with Pediatric Cancer

**DOI:** 10.3390/ijerph17061956

**Published:** 2020-03-17

**Authors:** Caitlin J. Cain, Andrea R. Meisman, Kirstin Drucker, Evrosina I. Isaac, Tanvi Verma, Jordyn Griffin, Jennifer M. Rohan

**Affiliations:** 1 Children’s Hospital of Richmond at Virginia Commonwealth University, Richmond, VA 23219, USA; Kirstin.Drucker@vcuhealth.org (K.D.); isaacei@vcu.edu (E.I.I.); tverma@mymail.vcu.edu (T.V.); Jordyn.Griffin@vcuhealth.org (J.G.); Jennifer.Rohan@vcuhealth.org (J.M.R.); 2 Georgetown University School of Medicine, Washington, DC 20007, USA; 3 Division of Adolescent and Transition Medicine, Cincinnati Children’s Hospital Medical Center, Cincinnati, OH 45229, USA; andrea.meisman@cchmc.org; 4 Virginia Commonwealth University School of Medicine, Richmond, VA 23219, USA; 5 Massey Cancer Center Virginia Commonwealth University, Richmond, VA 23219, USA

**Keywords:** adherence, electronic monitoring, cancer, metabolites, pharmacology

## Abstract

Previous research suggests that children and adolescents with acute lymphoblastic leukemia (ALL) and lymphoblastic lymphoma (LBL) often have difficulty adhering to complex treatment regimens during the maintenance phase of therapy. Measurement of treatment adherence can be done via objective (e.g., electronic monitoring (EM), pharmacological assays) or subjective methods (patient, parent, or physician reports). This paper provides an illustration of recommended strategies for comparing discrepancies between two objective measures of medication adherence (e.g., behavioral adherence using electronic monitoring versus pharmacological adherence using 6-mercaptopurine (6MP) metabolite data) within a relatively large cohort of pediatric patients with ALL or LBL (*N* = 139) who had longitudinal data for both measures of medication adherence over a 15-month period. Additionally, individual- and family-level factors such as gender, socioeconomic status, household environment, and dose intensity will be examined to identify possible sources of discrepancies between adherence measures. This information will provide practical advice for physicians, healthcare providers, and psychologists in identifying nonadherence and the caveats therein so patients achieve the best possible health outcomes.

## 1. Introduction: Importance of Using Novel Objective Measures of Medication Adherence

A pervasive obstacle in managing pediatric chronic illness, including pediatric cancer is treatment nonadherence [1,2,3,4,5,6,7,8,9]. Treatment nonadherence has the potential to cause significant negative health consequences for pediatric patients [10]. Previous research suggests that children and adolescents with acute lymphoblastic leukemia (ALL) and lymphoblastic lymphoma (LBL) often have difficulty adhering to complex treatment regimens during the maintenance phase of therapy—a phase where oral medication adherence is considered critical to survival and long-term successful outcomes for these patients [2,3,4,5,6,7,8]. During the maintenance phase of treatment, pediatric ALL and LBL patients must take a daily oral dose of 6-mercaptopurine (6MP), which is a chemotherapy medication prescribed to maintain disease remission. Patients are required to take this medication for a period of 2–3 years. Research has shown that adherence rates to 6MP ≤95% are often associated with increased risk of disease relapse [2,4].

Measurement of treatment adherence can be done via objective or subjective methods. This includes use of “direct measures” such as biological assays or clinician observation of medication ingestion and “indirect measures” such as self-report or parent report, which is a subjective method compared to more objective measures such as medical chart review documenting observed adherence rates, pill counts, or electronic monitoring (EM; e.g., medication event monitoring systems such as MEMs caps, MedSignals, SmartInhalers, blood glucose monitoring meters, continuous glucose monitors, continuous positive airway pressure machines (CPAP machine, which is a treatment method for patients who have sleep apnea or cystic fibrosis that helps keep their airways open, especially during sleep), etc.). Many studies have used physician, parent, or patient reported adherence measures or pill counts, which often overestimate adherence levels and are suboptimal measures to identify and target specific nonadherence patterns—especially as such data does not provide objective information, such as the date and time medication was administered or ingested [9,11]. While this critical information can be obtained by EM; EM is not necessarily a direct measure of oral medication adherence. Patients’ could remove medication from the bottle but not actually ingest it—necessitating the need for using objective indirect measures such as EM with direct measures such as metabolite assays to examine the specific influences of adherence on drug exposure, therapeutic efficacy, and clinical outcomes [2,9,11,12].

Metabolite concentration profiles of 6MP provide information regarding medication adherence for the time period immediately prior to the blood draw (i.e., a period of 5 days prior to the blood draw). When 6MP is ingested, it is metabolized into two metabolites: 6-thioguanine nucleotide (TGN) and 6-methylated mercaptopurine (MMP) with half-lives of about 5 days following the medication dose [13]. While there are individual differences in pharmacological patterns of 6MP adherence, there are three unique groups that have been described in the literature [2,14,15,16,17,18,19,20]: One group demonstrating nonadherence or poor bioavailability of the drug with low levels of both TGN and MMP. This group being most at risk of poor disease prognosis and disease relapse in children with ALL and LBL. While the other two groups demonstrate adherence with either a high TGN and low MMP profile or a low TGN and high MMP profile [2,14,15,16,17,18,19,20]. Metabolite concentration profiles can provide direct objective information regarding medication adherence and can be directly mapped on to electronic monitoring data [2,17].

In previous studies [2,17], researchers found that the majority of patients who showed low rates of medication adherence based on EM also were identified as nonadherent based on metabolite profiles with low levels of each metabolite. That said, there were subgroups of patients who demonstrated incongruent adherence profiles when examining their EM and pharmacological adherence results together. Little is known about what drives these incongruences between multiple objective measures of medication adherence; however, determining what specific factors may contribute to incongruences between novel measures of medication adherence will allow clinicians and healthcare providers to further enhance their patients’ adherence and ultimately their health outcomes, including decreased risk for disease relapse and decreased healthcare utilization. This paper provides an illustration of recommended strategies for comparing discrepancies between two objective measures of medication adherence (e.g., behavioral adherence using EM versus pharmacological adherence using 6MP metabolite data) using a large cohort of pediatric patients with ALL or LBL (*N* = 139) who had longitudinal data for both measures of medication adherence over a 15-month period. Additionally, individual- and family-level factors will be examined to identify possible sources of discrepancies between adherence measures. This information will provide practical advice for physicians, healthcare providers, and psychologists in identifying nonadherence and the caveats therein so patients achieve the best possible health outcomes, including disease remission and decreased risk of adverse events.

## 2. Comparison of Two Objective Measures of Medication Adherence: An Illustration in Pediatric Cancer Using Behavioral Adherence (EM) and Pharmacological Assays

While previous work has demonstrated the usefulness of using two novel objective measures of medication adherence to validate adherence findings [2,17], this becomes particularly important when there are instances when the metabolite profile and the EM adherence data are inconsistent. For instance, when examining the results of behavioral adherence versus pharmacological measures of medication adherence, there may be subgroups of patients who are identified as adherent based on their pharmacological adherence profiles (i.e., high TGN–low MMP profile, or a low TGN–high MMP profile), but their behavioral adherence profiles may demonstrate poor adherence to oral chemotherapy medication (i.e., medication adherence rates ≤95% for patients with ALL or LBL). There are a number of factors that could explain these differences, including patients who are slow metabolizers of the medication [16,21,22,23,24,25,26] or patients who take medication from another medication source besides their prescription bottle (e.g., patients who may have left a previous dose of medication on their bedroom dresser or in a secondary medication bottle). On the other hand, another subgroup of patients can be identified as nonadherent based on pharmacological profiles (i.e., low levels of both TGN and MMP) with behavioral adherence profiles suggestive of optimal adherence (i.e., rates of medication adherence ≥95%, which is indicative of the most favorable health outcomes for pediatric ALL and LBL [2,4]). Factors that may have contributed to such a discrepancy include patients who are high metabolizers of the medication or patients who removed the pill from the bottle, but never actually ingested the medication due to forgetting or intentional nonadherence [16,21,22,23,24,25,26].

Thiopurine methyltransferase (TPMT) is an enzyme that metabolizes 6MP into two active metabolites: methylated mercaptopurine (MMP) and thioguanine (TGN) [23,27]. TPMT activity is a genetic trait that is inherited from both biological parents. TPMT activity can be described as a genotype (e.g., homozygous deficient, heterozygous, or homozygous wild type). TPMT activity is an important measure in pediatric cancer because it can affect how a patient metabolizes 6MP, which could potentially influence the metabolite profile group membership for that patient. If patients are adherent to their 6MP medication, patients with a “homozygous deficient” genotype will often present with extremely high concentrations of TGN metabolites and little-to-no MMP metabolites. Patients with a “heterozygous” genotype who are taking 6MP medication as prescribed will often present with high concentrations of TGN metabolites and low levels of MMP metabolites. Patients with “homozygous/wild-type” genotype will present with low levels of TGN and high levels of MMP if taking their medication as prescribed [23,27].

This commentary will further examine potential causes of discrepancies between multiple objective adherence measures using pediatric cancer as an example. Patient age, average 6MP behavioral adherence, 6MP absolute metabolite levels, duration of cancer diagnosis and maintenance therapy, patient gender, patient ethnicity/race, household composition (one- vs. two-parent households), annual household income, medication administration patterns, 6MP dose recommendations, and thiopurine methyltransferase (TPMT) activity will be evaluated. The relationship between incongruent adherence results and health outcomes (disease relapse, death) will also be examined. This commentary is an extension of a previously published research study that examined the relationship between two objective measures of medication adherence: behavioral adherence (MEMs data) and pharmacological adherence profiles (6MP metabolites) [2].

Although the previous work showed a strong relationship between these two objective measures of medication adherence [2], there were subgroups of patients who demonstrated incongruent profiles (i.e., optimal adherence on one measure and suboptimal adherence on the other measure). This paper further examines the incongruences between these two measures of medication adherence described in the previous studies [1,2] in an effort to further understand the subgroups of patients who demonstrated discrepancies between multiple medication adherence measures. Previous studies were published from this dataset describing baseline adherence patterns [1] and the validation of the behavioral adherence data with the pharmacological adherence data [2].

To further understand the demographic and medical characteristics of the original study sample, a brief overview of the original study is described here. Please see prior publications for a full review of the study, including demographic and medical characteristics [1,2]. Study participants in the original study included 139 patients ages 7–19 years at baseline who were diagnosed with ALL (*n* = 133, 95.7%) or LBL (*n* = 6, 4.3%). All patients in the original study were taking 6MP medication daily and were in maintenance therapy for pediatric cancer. The original study was approved by an Institutional Review Board. Adherence and medical data were collected as part of the 15-month longitudinal study. Medical charts were reviewed at quarterly intervals across 15 months using standardized forms to obtain information including prescribed treatment regimens (medication type, dose, and timing of administration) and disease relapse and mortality. Information regarding the prescribed treatment regimen was used to operationalize nonadherence (e.g., discrepancy between the daily dose of 6MP versus what the patient took as measured by electronic monitoring and pharmacological data).

### 2.1. Behavioral Adherence Data Was Captured through Electronic Monitoring of 6MP Adherence Across 15-Months Using the Medication Event Monitoring System (MEMS^®^) from the AARDEX Corporation, Palo Alto, CA

The MEMS^®^ system is analogous to a commonly used prescription bottle, but the cap of the device contains a micro-electronic chip that registers dates and times when the bottle is opened and closed. Patients and families were aware of adherence monitoring but were not given feedback regarding their adherence; they were also instructed to take 6MP only from the MEMS^®^ bottle for the duration of the study. A standardized form was administered at quarterly intervals to capture information regarding extra openings, refills, possibility of using alternative methods of medication adherence, and periods of nonuse. For the purposes of this commentary, behavioral medication adherence for the 5 days prior to the blood draw was used given the half-life of 6MP is only 5 days [2,23,27].

### 2.2. Pharmacological Measures of 6MP Adherence Were Measured Using Metabolite Levels of 6MP

Blood samples were collected at quarterly intervals across 15 months. Metabolite levels at each time point, the last dosage amount, and the date and time of the last dose were mapped on and verified with electronic monitoring data. Metabolite profiles were created using cluster analysis to document potential nonadherence or suboptimal therapy (low levels of both metabolites) and adherent metabolite profiles (i.e., inverse relationship between TGN and MMP). Please see published work by Rohan and colleagues describing the metabolite profiles and behavioral adherence data [2].

The present work described discrepant profiles of two objective measures of medication adherence. For the purposes of this commentary, all time points from baseline to 15 months were collapsed together to examine incongruent adherence profiles across the 15-month period (*n* = 192 samples). Patients’ 6MP medication adherence was categorized into two groups based on the results of the behavioral and adherence measures: (1) optimal behavioral adherence (behavioral adherence rates ≥95%) vs. poor 6MP adherence based on pharmacological metabolite profiles (low–low levels of both metabolites); and, (2) poor behavioral adherence (behavioral adherence rates <95%) vs. adherent metabolite profiles (inverse relationship between MMP–TGN profiles).

Demographic and medical characteristics for the two profiles are shown in Table 1. In the present study, 191 samples collected across the 15-month period demonstrated discrepancies between behavioral and pharmacological adherence measures. When comparing the two groups, patients (*n* = 111; 58.1%) with optimal behavioral adherence (adherence rates >95%), but nonadherent metabolite profiles (low–low metabolites) had mean 5-day adherence rates of 100% across 15 months compared to mean 5-day adherence rates of 50% in the poor behavioral adherence (adherence rates <95%) and adherent metabolite profile group (*n* = 80, 41.9%).

The number of two-parent households (76.6%) also was higher in the optimal behavioral adherence–nonadherent pharmacological profile compared to the poor behavioral adherence–adherent pharmacological profile (65%). Additionally, those with 95% behavioral adherence but suboptimal metabolite levels reported higher annual income (57.3% reported income ≥$49,000) compared to the <95% behavioral adherence–adherent pharmacological profile who reported lower annual incomes (55.9% reported income <$49,000).

While it would be expected that a higher percentage of patients with lower behavioral adherence rates but adherent metabolite profiles would report potentially using alternative medication systems instead of the primary prescription bottle, there was a relatively equal percentage of patients who denied using other medication administration options (57.5%) relative to those who reported possibly using another system (e.g., 42.5% reported taking a dose outside of the prescription bottle, using an extra prescription bottle, etc.).

One possible explanation for high behavioral adherence and low metabolites may be related to dosing recommendations—i.e., receiving lower doses that what was recommended based on body surface area (BSA). In the present sample, 88.3% of patients in this group were prescribed 6MP doses within or above recommended dosages based on body surface area; whereas, 11.7% were prescribed doses below recommendations. This finding was not observed in the low behavioral adherence–adherent metabolite profile group where most patients (98.8%) had dosing within or above dosage recommendations. Finally, there were minimal differences observed between the two groups when looking at disease relapse and mortality rates.

## 3. Conclusion: Importance of Using Multiple Objective Measures of Medication Adherence

The mere existence of discrepant cohorts when examining multiple objective measures of medication adherence merits further consideration. One possible explanation for the behavioral adherent–pharmacological nonadherent cohort is dose intensity. There may be discrepancies in prescribed dosing versus dosing recommendations, which could impact the metabolite levels of 6MP because patients are not receiving the recommended dosing and as such are presenting with subtherapeutic metabolite levels. In the present sample, 11.7% of patients with this profile had 6MP doses prescribed below recommended dosages even after adjusting for weight changes over time. It could be possible that 6MP doses fell below standard dosage recommendations due to deliberate dose adjustments related to absolute neutrophil count. This should be explored in future studies. In clinical experience, it is not uncommon for parents or patients to report that they removed a pill from the medication bottle, but the patient never actually ingested the pill due to forgetting, deciding to take later but then losing the medication or forgetting to take it, or intentional nonadherence (e.g., worry about side effects, feeling different from peers, etc.). This could explain why behavioral adherence rates were >95%, but the patient had low levels of metabolites.

The second cohort of interest in this commentary is the poor behavioral adherence–adherent metabolite profile. Mean adherence rates for this profile were 50% with moderate variability observed within the cohort (SD = 30.52). It is notable that 100% of patients in this cohort had adherence rates <85%. Bhatia and colleagues [4] recommended that for optimal health outcomes for pediatric ALL and LBL patients’, including decreased risk for disease relapse and mortality, 6MP medication adherence should be 95% or higher across the treatment period. As indicated above, patients’ with this profile reported lower annual incomes, had a higher frequency of single-parent households (35% compared to 23.4%), and had a relatively equal distribution between reporting that they always used the same prescription bottle (57.5%) versus reporting that there is a possibility that the patient took medication from another source (42.5%).

The results presented within the previous work [2] as well as this commentary underscores the relative importance of using multiple objective measures of medication adherence to understand the biopsychosocial elements of medication taking and its effects on health outcomes. Both metabolite profiles and behavioral adherence measures provide unique and much needed information. While pharmacological adherence measures provide objective information on whether the medication was ingested, behavioral adherence measures such as electronic monitoring provide daily information regarding the date/time medication was removed from the bottle, thus directly complementing one another. It may seem that in clinical practice there are more practical ways to gauge adherence. For example, with 6MP medication, dosing is titrated to a therapeutic level based on leukocyte count, and a leukocyte count in the desired range indicates adequate levels for treatment. Simply titrating a medicine does not address the underlying issue of why a patient may or may not be adherent. Thus, there is utility in understanding and considering the basis for why a patient may seem and be labeled as “nonadherent” or “noncompliant” by providers when in reality they are compliant with their medications. Such a consideration of adherence and the psychology therein is applicable not only to chemotherapy medications but also to any medication taken long term that works best when at adequate, sustained dosing levels. Moreover there is the central component of genetics in drug metabolism, in this instance illustrated by TMPT phenotypes, which may affect pharmacological adherence profiles [23]. Future research should examine these incongruent profiles further, including statistical analysis of outcomes in these disparate adherence groups. In the interim, it is important for providers to speak directly to patients and families when incongruences exist and when nonadherence is suspected, to further understand and identify these unique groups of patients with an emphasis on reducing adverse outcomes and maximizing adherence and health behaviors over time.

## Figures and Tables

**Table 1:** Demographic and medical characteristics of incongruent adherence profiles across 15 months.

Cohort Demographics	≥95% Beh Adh/Lo–Lo Met Grp (*n* = 111)	<95% Beh Adh/Adh Met Grp (*n* = 80)
Patient age at baseline (years), M ± SD	12.36 ± 3.34	12.32 ± 3.65
Average 6MP Behavioral Adherence (5-day), M ± SD	100.00 ± 0.00	50 ± 30.52
6MP Metabolite Levels, Absolute Values		
TGN (Absolute Value), M ± SD	446.34 ± 189.51	708.37 ± 415.81
MMP (Absolute Value), M ± SD	7029.48 ± 5092.65	23,748.94 ± 12,819.77
Duration of Cancer Diagnosis at Baseline (Years), M ± SD	1.33 ± 0.35	1.24 ± 0.34
Duration of Maintenance Treatment at Baseline (Months), M ± SD	17.58 ±10.30	14.61 ± 9.91
Child’s Gender, *n* (%)		
Male	74 (66.7)	52 (65)
Female	37 (33.3)	28 (35)
Child’s Ethnicity/Race, *n* (%)		
Non-Hispanic, Caucasian	67 (60.4)	41 (51.3)
Non-Hispanic, Minority	17 (15.3)	10 (12.5)
Hispanic	27 (24.3)	29 (36.3)
Household Composition, *n* (%)		
One-caregiver household	26 (23.4)	28 (35)
wo-caregiver household	85 (76.6)	52 (65)
Total Annual Household Income (Before Taxes), *n* (%)		
<$18,745	19 (17.3)	18 (23.4)
$18,745–$32,874	15 (13.6)	18 (23.4)
$32,875–$48,999	13 (11.8)	7 (9.1)
$49,000–$72,999	18 (16.4)	9 (11.7)
$73,000–$126,500	26 (23.6)	20 (26.0)
>$126,000	19 (17.3)	5 (6.5)
Possibility of Different Medication Administration Method Used		
No	80 (72.1)	46 (57.5)
Yes	31 (27.9)	34 (42.5)
6MP Dose Recommendations		
Prescribed 6MP Dose Within/Above Dose Recommendations	98 (88.3)	79 (98.8)
Prescribed 6MP Dose Below Dose Recommendations	13 (11.7)	1 (1.3)
TPMT Genotype		
Heterozygote	12 (11.8)	10 (12.5)
Wild type	90 (88.2)	70 (87.5)
Patient Relapsed		
No	103 (94.5)	74 (94.9)
Yes	3 (5.5)	4 (5.1)
Patient Deceased		
No	109 (100)	77 (98.7)
Yes	0 (0)	1 (1.3)

Note: Column 1 represents the cohort with behavioral adherence (Beh Adh) rates ≥95% and low TGN-low MMP metabolites (Lo–Lo Met Grp). Column 2 represents the cohort with behavioral adherence (Beh Adh) rates <95% and adherent metabolites profiles (Adh Met Grp), which were the metabolite profiles indicating high TGN–low MMP levels or low TGN–high MMP levels.
